# Supplementary material for: A UMLS-based spell checker for natural language processing in vaccine safety
Source: BMC Med Inform Decis Mak. 2007 Feb 12;7:3. doi: 10.1186/1472-6947-7-3 (PMC1805499; doi:10.1186/1472-6947-7-3)
Supplement: Additional file 13 — RAP application directory. Sets up directory for RAP (RDF application for PHP) source code [file 1472-6947-7-3-S13.gz › rap/doc/phpdoc/index_memmodel.html]

xml version="1.0" encoding="iso-8859-1"?


RAP - Rdf API for PHP V0.9


<H2>Frame Alert</H2>
<P>This document is designed to be viewed using the frames feature.
If you see this message, you are using a non-frame-capable web client.</P>
